# A Retrospective Assessment of HIV Prevalence in the Central Black Sea Region of Türkiye

**DOI:** 10.3390/tropicalmed11070177

**Published:** 2026-06-27

**Authors:** Mehmet Hakan Taskin, Sule Ozturk, Esra Tas, Reyhan Caliskan, Ergin Kariptas, Ecem Dilara Aygun, Seda Gozel, Zafer Yazici, Ahmed Eisa Elhag

**Affiliations:** 1 Department of Medical Microbiology, Faculty of Medicine, University of Samsun, 55080 Samsun, Türkiye; mehmet.taskin@samsun.edu.tr (M.H.T.); esra.tas@samsun.edu.tr (E.T.); reyhan.caliskan@samsun.edu.tr (R.C.); ergin.kariptas@samsun.edu.tr (E.K.); 2 Department of Public Health, Faculty of Medicine, University of Samsun, 55080 Samsun, Türkiye; sule_ozt@hotmail.com; 3 Department of Virology, Faculty of Veterinary Medicine, Ondokuz Mayis University, 55139 Samsun, Türkiye; ecemdilaraa@gmail.com (E.D.A.); seda.gozel@omu.edu.tr (S.G.); 4 Institute of Biochemistry and Biophysics, Polish Academy of Sciences, 02-106 Warsaw, Poland; 5 Department of Preventive Medicine and Clinical Studies, Faculty of Veterinary Sciences, University of Gadarif, Al Qadarif 32211, Sudan

**Keywords:** anti-HIV, epidemiology, flow cytometry, HIV RNA, human immunodeficiency virus, prevalence

## Abstract

Background: HIV has been a major global health issue since the 1980s. The proportions of diagnosed and undiagnosed infections are important indicators of HIV control. This study investigated the prevalence and characteristics of HIV based on blood samples from patients presenting for diagnosis and treatment at a tertiary healthcare hospital in the Central Black Sea Region over a four-year retrospective period. Methods: We retrospectively evaluated 271,367 samples submitted for anti-HIV serology testing for pre-operative screening or clinical suspicion between 2020 and 2023. HIV-1/2 antibodies and p24 antigen were screened using chemiluminescence microparticle immunoassay (CMIA), and reactive samples were confirmed by real-time RT-PCR. HIV RNA results and immunological parameters were also analyzed. Results: Among 271,367 samples, 694 were HIV-positive, yielding a prevalence of 0.25%. Of 2207 individuals tested for HIV RNA, 699 (31.7%) were positive. HIV RNA positivity was higher in men than women (35.5% vs. 18.6%). Absolute CD4+ T-cell counts were similar between genders, while the CD4/CD8 ratio was significantly higher in women. Both CD4+ T-cell counts and CD4/CD8 ratios increased significantly after three months of treatment. Conclusions: This study demonstrates a low but increasing HIV seroprevalence in the region, with a clear predominance among males and young adults. The findings also reflect effective viral suppression among treated patients and significant immunological recovery after therapy, providing important regional data to guide HIV monitoring and public health interventions.

## 1. Introduction

Human immunodeficiency virus (HIV) is a member of the genus Lentivirus in the subfamily Orthoretrovirinae within the family Retroviridae. It severely damages the immune system and causes acquired immunodeficiency syndrome (AIDS), which leads to a range of potentially life-threatening infections and diseases. Since the recognition of AIDS in 1981 and the discovery of HIV as the causative agent, the global HIV/AIDS pandemic remains one of the most significant public health threats to humanity. Despite a decline in both new HIV infections and HIV-related deaths due to remarkable scientific progress, antiretroviral therapy (ART), effective HIV prevention, and innovative treatment models, more than 88 million people have been infected with HIV, and approximately 42 million people have died due to HIV during this time [1,2,3]. It has been reported that the number of people infected with HIV globally was 39.9 million by the end of 2023, 65% of whom were in African countries. In 2023, more than 630,000 people died from AIDS-related diseases, while the number of newly acquired HIV infections was estimated to be 1.3 million worldwide [1,3].

HIV targets CD4+ T-helper (Th) cells, which are the key regulators of humoral and cellular immune responses in humans. The virus infects these cells, leading to their continuous loss [4,5]. A decline in CD4+ T-helper cells is a sign of a weakened immune system and also indicates progression to AIDS. During this process, HIV compromises the immune system and paves the way for a variety of opportunistic infections and diseases that can lead to death [4].

Even with effective ART leading to sustained HIV RNA suppression and adequate CD4+ T-cell recovery, people living with HIV generally do not achieve the same life expectancy as HIV-negative individuals, largely due to the ongoing risk of both AIDS-related and non-AIDS-related comorbidities. Compared to those without HIV, persons with HIV (PWH) on ART who have undetectable HIV RNA and sufficient CD4+ T cell counts are more likely to experience a number of non-AIDS comorbidities and may live shorter lives. All participants received antiretroviral therapy (ART) in accordance with the World Health Organization (WHO) guidelines, which recommend initiating treatment regardless of CD4+ T cell count and continuing lifelong therapy to achieve and maintain viral suppression.

Recent longitudinal data indicate that even in virologically suppressed patients with adequate CD4+ T-cell recovery, a persistently low CD4/CD8 ratio and elevated CD8+ T-cell counts remain independent predictors of both AIDS-related and non-AIDS-related morbidity and mortality, underscoring their prognostic value beyond traditional markers [6]. However, due to their associations with the potential mechanisms underlying the development of AIDS and non-AIDS-related comorbidities, the CD4/CD8 ratio and CD8+ T cell counts have gained recognition as novel indicators in recent years [6].

In Türkiye, HIV/AIDS was included in the list of notifiable diseases in 1985, and national surveillance has been conducted since the first case was officially recorded that same year. According to the statistical report of the Ministry of Health, published by the General Directorate of Public Health (Halk Sağlığı Genel Müdürlüğü, Bulaşıcı Hastalıklar Daire Başkanlığı), from 1985 to the end of 2024, the number of confirmed HIV-positive individuals and AIDS cases in Türkiye were 45,835 and 2438, respectively, reported separately because AIDS cases are a subset of HIV infections and combining them would result in double counting [7]. This official record is based on the document “Ek HIV-AIDS İstatistikleri,” accessed on 7 November 2024, which provides nationwide cumulative data and confirms that the first documented HIV/AIDS case in Türkiye occurred in 1985 [7].

In this study, we aimed to retrospectively evaluate the results of anti-HIV tests performed in a tertiary referral hospital in Samsun province, in the Central Black Sea Region of Türkiye. In addition, we compared pre- and post-treatment absolute CD4+ T-cell count and CD4/CD8 ratios obtained using flow cytometry.

## 2. Materials and Method

### 2.1. Study Setting and Population

This study was conducted at Samsun Training and Research Hospital, a tertiary-level teaching and research hospital affiliated with the Faculty of Medicine at Samsun University (SAMÜ), located in Samsun, the largest city on the north coast of Türkiye, with a population exceeding one million. The hospital provides healthcare services to more than 900,000 patients annually from Samsun and surrounding provinces, functioning as a major referral center in the Central Black Sea Region. Owing to its advanced laboratory infrastructure and specialized infectious diseases units, the hospital serves as a regional hub for HIV screening, diagnosis, and treatment follow-up, ensuring that the data reflect a broad cohort representative of regional HIV epidemiology and antiretroviral therapy (ART) outcomes.

We retrospectively evaluated anti-HIV serology and HIV RNA PCR results from 271,367 unique individuals who attended the hospital between 2020 and 2023 for routine pre-operative screening or due to clinical suspicion or diagnosis of HIV/AIDS. Data were analyzed by age, gender, and year of presentation, with only one result included per individual. For confirmed HIV-positive cases, absolute CD4+ T-cell counts and CD4/CD8 T-cell ratios were assessed before and after treatment, as key indicators of immune status, disease monitoring, and opportunistic infection risk.

### 2.2. Ethical Approval

This retrospective study was conducted following approval from the Non-Interventional Clinical Research Ethics Board of Samsun University (decision number: GOKAEK 2024/8/14; approval date: 17 April 2024). The data were accessed for research purposes on 20 April 2024. The authors had access to information that could identify individual participants during or after data collection. All procedures were conducted in accordance with the principles of the Declaration of Helsinki.

### 2.3. Detection of HIV

The Alinity HIV Ag/Ab combo test (Abbott Laboratories, Chicago, IL, USA), a chemiluminescent microparticle immunoassay (CMIA), was used for the simultaneous qualitative detection of HIV p24 antigen and antibodies to HIV types 1 and/or 2 in human sera and plasma specimens on the Abbott Alinity Analyzer (Abbott laboratories, IL, USA), following the manufacturer’s instructions. The specificity and sensitivity of this method were reported by the manufacturer as 99.2% and 100%, respectively, with a 95% confidence interval.

### 2.4. Real-Time PCR Assay for HIV

For confirmation and quantification of HIV in positive samples, HIV-RNA PCR was performed using the COBAS AmpliPrep/COBAS TaqMan HIV-1 test (Roche, Mannheim, Germany), an in vitro nucleic acid amplification test for the quantification of HIV-1 RNA. The process was carried out on the COBAS AmpliPrep Instrument (Roche, Mannheim, Germany) for automated specimen processing and the COBAS^®^ TaqMan Analyzer (Roche, Mannheim, Germany).

HIV-RNA testing was performed both for the confirmation of anti-HIV–positive cases detected by CMIA in our center and for the monitoring of patients already known to be HIV positive, including those diagnosed in other healthcare facilities and referred to our hospital for confirmation or follow-up, as well as patients under regular antiretroviral therapy (ART) monitoring in our clinic. In selected cases with negative serology but clinical suspicion of acute HIV infection or high-risk exposure, HIV-RNA testing was also performed simultaneously with CMIA. The CMIA method used in this study detects HIV-1/2 antibodies and p24 antigen simultaneously but does not differentiate between HIV-1 and HIV-2. Specific testing for HIV-2 was not performed, as WHO data (Global HIV, Hepatitis and STIs Programs, HIV Data and Statistics) indicate that HIV-2 infection is extremely rare outside of West Africa.

### 2.5. Flow Cytometry Analysis

To determine T-cell subsets in peripheral blood samples from HIV-positive patients, flow cytometry was performed as previously described [8]. Initially, samples were analyzed using a CD45 versus side scatter (SSC) dot plot to precisely identify the lymphocyte population, characterized by high CD45 expression and low cellular granularity (SSC). This approach allowed for the systematic exclusion of monocytes, cellular debris, and unlysed red blood cells from the analysis. Further analysis was conducted on the CD3+ T-cell population in the CD3–SSC plot, with T-cell differentiation patterns assessed in CD3+CD4+ and CD3+CD8+ plots. A CD4-FITC/CD8-PE/CD19-ECD/CD3-PC7/CD45-APC antibody combination was used for flow cytometric evaluation. For each sample, a minimum of 20,000 events was acquired in the initial lymphocyte gate to ensure statistical robustness. Percentages of CD3+CD4+ and CD3+CD8+ lymphocytes were determined using precise lymphocyte scatter gates and two-color immunofluorescence, as described previously [8]. Data were processed and analyzed using Kaluza analysis software (version 2.1).

For absolute counts, 100 μL of Flow-Count fluorospheres (Beckman Coulter, FL, USA) was added to each lysed sample before analysis. Absolute CD4 and CD8 T-cell counts were automatically calculated by the System II software (version 4.0) using the formula:

$$N=\frac{\mathrm{CD}3+\;\mathrm{CD}4+\;\mathrm{or}\;\mathrm{CD}3+\;\mathrm{CD}8+\mathrm{lymphocytes}\;\mathrm{counted}}{\mathrm{fluorospheres}\;\mathrm{counted}\times \mathrm{fluorospheres}/\mathrm{microliter}}$$

### 2.6. Statistical Analysis

Statistical analyses were performed using SPSS software (version 26.0; IBM Corp., Armonk, NY, USA). The normality of the data was evaluated using the Shapiro–Wilk test. Descriptive statistics were expressed as numbers and percentages for categorical variables. For continuous variables, data were presented as mean ± standard deviation or median and interquartile range (IQR, Q1–Q3), depending on the distribution.

To compare independent groups, the Mann–Whitney U test was used. For the comparison of absolute CD4+ T-cell counts and CD4/CD8 ratios before and after the initiation of ART in the same individuals, the paired-samples t-test or Wilcoxon signed-rank test was employed. Categorical data were compared using the Pearson chi-square test or Fisher’s exact test. For all analyses, a *p*-value < 0.05 was considered statistically significant.

Multivariate logistic regression analysis was performed to identify independent predictors of HIV positivity and HIV-RNA positivity. Variables included age, gender, and year of testing. Adjusted odds ratios (ORs) with 95% confidence intervals (CIs) were calculated.

## 3. Results

In this study, we retrospectively evaluated the results of 271,367 patient samples from individuals who attended the hospital between 2020 and 2023 for HIV/AIDS testing. The median age of the individuals was 47.0 years (Q1–Q3: 31.0–63.0; Q1–Q3 indicates the interquartile range, i.e., the middle 50% of the data between the 25th and 75th percentiles). Of the total individuals, 56.2% (*n* = 152,471) were women and 43.8% (*n* = 118,896) were men (Table 1). The annual distribution of the analyzed samples was as follows: 43,021 (15.9%) in 2020, 54,838 (20.2%) in 2021, 77,586 (28.6%) in 2022, and 95,922 (35.3%) in 2023.

A total of 694 samples were anti-HIV positive, corresponding to an overall prevalence of 0.25% (Table 1). Annual HIV positivity rates were 0.2% in 2020 (*n* = 106), 0.3% in 2021 (*n* = 153), 0.3% in 2022 (*n* = 214), and 0.2% in 2023 (*n* = 221). Anti-HIV positivity showed no significant correlation with year (*p* = 0.175) (Table 1). Among anti-HIV–positive cases, 598 (0.5%) were men and 96 (0.1%) were women, with a significantly higher rate in men (*p* < 0.001) (Table 1). The median age of anti-HIV–positive patients (38.0 years) was significantly lower than that of negative patients (47.0 years) (*p* < 0.001).

Between 2020 and 2023, HIV-RNA testing was performed in a total of 2207 patients (one sample per patient) to confirm HIV and determine viral loads (Table 2). This group included all anti-HIV–positive cases newly identified in our center (*n* = 694) for confirmation, as well as patients diagnosed as anti-HIV-positive in other centers and referred to our hospital for confirmation or follow-up, and individuals already under ART in our clinic who underwent periodic HIV-RNA monitoring. Additionally, in selected cases with clinical suspicion of acute HIV infection or a history of high-risk exposure, HIV-RNA testing was performed simultaneously with CMIA (Table 2).

Of the 2207 individuals tested, 1707 (77.3%) were men and 500 (22.7%) were women. A total of 699 (31.7%) were HIV-RNA positive. The positivity rate was significantly higher in men (35.5%, 606/1707) than in women (18.6%, 93/500) (*p* < 0.001). The majority of HIV-RNA–positive cases were men (86.69%, 606/699) (Table 2). The distribution of samples from individuals tested for HIV-RNA by year was 435 (19.7%) in 2020, 385 (17.4%) in 2021, 592 (26.8%) in 2022, and 795 (36.1%) in 2023 (Table 2). HIV-RNA positivity was highest in 2023 (73.5%), followed by 2020 (73.1%), 2022 (64.5%), and 2021 (58.2%) (*p* < 0.001) (Table 2). Among the 22 individuals under 18 years of age who underwent HIV-RNA testing, one was positive (Table 2).

The median age of HIV-RNA–positive cases was 38.0 years, significantly lower than that of HIV-RNA–negative cases (41.0 years) (*p* < 0.001). Among HIV-RNA–positive patients, the median age of men was 38.0 years, which was higher than that of women (36.0 years).

To identify independent predictors of HIV-RNA positivity, a multivariate logistic regression analysis was performed, including age, gender, and year of testing (Table 3). Male gender was independently associated with a higher likelihood of HIV-RNA positivity (OR: 2.41, *p* < 0.001). In contrast, increasing age was associated with a decreased risk (OR: 0.97 per year, *p* < 0.001). The year of testing was not a significant predictor after adjustment (*p* = 0.174).

We also presented data on CD4+ counts and CD4+/CD8+ ratios obtained by flow cytometry in HIV-RNA–positive individuals and analyzed them by gender, before and after ART (Table 4 and Table 5). As shown in Table 4, the median CD4+ count was similar in men and women (*p* = 0.960), whereas the CD4+/CD8+ ratio was significantly higher in women compared to men (*p* < 0.001).

In Table 5, T cell parameters of HIV-RNA–positive individuals were compared before and after ART as overall and per gender results. Before treatment, absolute CD4+ T-cell counts decreased in 136 patients (19.5%), and CD4+/CD8+ ratios decreased in 84 patients (12.0%). However, a significant increase in CD4+ T cell count was observed following ART (from 465.0 cells/µL to 537.0 cells/µL; *p* < 0.001). Similarly, the CD4+/CD8+ ratio also increased after treatment (from 0.51 cells/µL to 0.70 cells/µL; *p* < 0.001). After ART, CD4+ T-cell counts and CD4+/CD8+ ratios increased significantly in men (*p* < 0.001 for both), whereas the increases observed in women were not statistically significant (*p* = 0.060 and *p* = 0.184, respectively).

A Spearman correlation analysis was performed to evaluate the relationship between absolute CD4+ T-cell counts and CD4+/CD8+ ratios in 699 patients (individuals) followed at our hospital. A significant positive correlation was observed between pre-treatment and post-treatment absolute CD4+ T-cell counts (r = 0.634, *p* < 0.001) and between pre-treatment and post-treatment CD4+/CD8+ ratios (r = 0.787, *p* < 0.001). In contrast, there was a low, non-significant positive correlation between absolute CD4+ T-cell counts and CD4+/CD8+ ratios before treatment (r = 0.026, *p* = 0.566), whereas a low, non-significant negative correlation was found between these parameters after treatment (r = −0.063, *p* = 0.158).

## 4. Discussion

The large testing volume in our research (271,367 samples) reflects both the high level of HIV awareness in the region and the substantial screening capacity of the involved hospital. However, due to the retrospective design, some patient records were incomplete, and certain laboratory and demographic data were missing. This constitutes an inherent limitation of retrospective, hospital-based evaluations. However, our study presents important data on the current epidemiological situation in the region by evaluating four years of HIV screening data and immunological parameters from 2020 to 2023 at a tertiary referral hospital in the Central Black Sea Region. The HIV seroprevalence rate of 0.25% found in this study implied that Türkiye was a low-HIV-prevalence country, while also revealing significant regional differences as compared globally. It has been previously reported that the adult HIV prevalence globally is approximately %0.7 [0.6–0.8], with higher rates particularly in Sub-Saharan Africa (SSA), whereas relatively lower rates in certain parts of Europe and the Middle East [3,7,9].

In contrast, new HIV infections have increased by 116% in Eastern Europe and Central Asia since 2010, highlighting that epidemiological dynamics can vary significantly by region [3,9]. This increase was reported to be particularly among the young adult population, and 2023 data from Europe show that a substantial majority of new HIV cases were predominantly observed in the 25–34 age group [10]. This finding was consistent with our results, which showed a rising trend in the young age group. Furthermore, several factors, including tourist mobility, migration, sociocultural behavioral differences, and access to testing, may play a role as key determinants of HIV prevalence. In our study, the rise in the number of positive cases, parallel to the increase in the number of samples tested, particularly the highest testing volume recorded in 2023, indicates an increase in clinical burden and awareness in the region. The increase in prevalence from 0.14% reported in our previous study to 0.25% in this work, along with the expanded four-year cohort, highlights the need for continuous monitoring of case dynamics in the region.

The 31.7% HIV RNA positivity rate detected in individual samples may reflect the effective application of antiretroviral therapy (ART) in the patient cohort. This is mainly because the study population included patients who were clinically followed and receiving ART. Therefore, it can be concluded that HIV RNA-negative individuals among anti-HIV-positive individuals represent successful viral suppression. This finding is consistent with the UNAIDS 95-95-95 goals. In 2023, nearly 1.3 million people were newly infected with HIV, which was more than three times higher than the UNAIDS 2025 target of fewer than 370,000 new infections [3]. Furthermore, an increase in the number of new HIV infections in Eastern Europe and Central Asia, Latin America, the Middle East, and North Africa was noteworthy in 2023 [3].

The percentage of people living with HIV (PLHIV) who are receiving ART is increasing due to global prevention and intervention efforts. Rapid initiation of ART immediately after HIV diagnosis is considered one of the most effective public health strategies for two main reasons: (i) ART reduces HIV transmission in serodiscordant couples by 93–96% and accelerates viral load suppression, thereby limiting further spread of the virus [11,12], and (ii) early initiation of therapy improves health-related quality of life and decreases morbidity and mortality among PLHIV [11].

As a result, in many countries, the prevalence of HIV has either declined or stabilized. According to the global HIV prevention targets for 2030, 95% of PLHIV should be aware of their status, 95% of those aware should be on treatment, and 95% of those on treatment should achieve viral suppression. In Türkiye, modeling studies suggest that achieving these 95-95-95 targets would lead to a substantial reduction in HIV incidence by 2030 [12]. By the end of 2023, an estimated 39.9 million (36.1–44.6 million) individuals worldwide were living with HIV [13]. According to WHO, the global prevalence among adults aged 15–49 years was 0.6% (0.6–0.7%) [13]. In 2023, 47 countries reported a total of 112,883 new HIV diagnoses, of which 24,731 were from European Union countries [14]. This corresponds to a crude diagnosis rate of 12.7 per 100,000 population, representing a 2.4% increase compared with 2022, but still lower than the pre-pandemic levels of 2019 [14]. Notably, 21 of 47 countries reported increases in 2023, with Lithuania, Malta, Montenegro, Azerbaijan, Finland, Iceland, Ireland, and Kazakhstan recording their highest figures in the past decade [14].

HIV-related testing in Türkiye began in 1986, following the first diagnosis of the disease. In 1987, testing became mandatory for blood, tissue, and organ donations, registered sex workers, and prior to major surgical procedures. In recent years, the prevalence of HIV infection in Türkiye has increased markedly. Several factors may have contributed to this rise: the predominantly young population structure, the rapid influx of immigrants from Syria and other countries, and limited opportunities for public education on communicable diseases. In addition, Türkiye’s growing tourism industry, coupled with the increasing number of Turkish citizens traveling abroad for work and leisure, has enhanced cross-border mobility. Intravenous drug use has also become more common, which presents another risk factor. According to data from the Ministry of Health, between 1985 and 7 November 2024, a total of 45,835 HIV-positive cases and 2438 AIDS cases were reported. Of these, 81.8% were men, 18.2% women, and 16.1% foreign nationals. The most frequently affected age groups were 25–29 and 30–34 years, respectively [7].

Samsun Training and Research Hospital is one of the leading healthcare centers in the region. With its wide patient portfolio and successful initiatives, it serves not only the city of Samsun but also the surrounding provinces. In this study, we retrospectively evaluated a total of 271,367 anti-HIV tests performed in our molecular diagnostic laboratory between 2020 and 2023. These years coincided with the COVID-19 pandemic in Türkiye, during which strict curfews were implemented on many days. PLHIV, who often have comorbidities and may be at higher risk for COVID-19–related complications, were significantly affected by the pandemic, which also had repercussions on equitable access to treatment and prevention.

According to the 271,367 anti-HIV test results from our hospital over this 4-year period, the overall prevalence was 0.25%. In our previous study, we reported an HIV prevalence of 0.14% between January and September 2022 [8]. Thus, the prevalence showed an increase over the 4-year period. Among all individuals tested, 56.2% were women, and the mean age was 47 years. However, anti-HIV test positivity was significantly higher among men (Table 2). The mean age of individuals with positive anti-HIV results was lower than that of those with negative results. No significant difference was found in terms of age or gender among individuals tested for HIV RNA. When the age distribution of HIV RNA–positive individuals was examined, the highest proportion was in the 25–34 age group (37.4%). This distribution is consistent with that reported for Türkiye overall.

When the results were analyzed by year, the majority of HIV RNA tests were performed in 2023. In our study, the HIV RNA positivity rate among the 2207 individuals tested was 31.7% (*n* = 699). This relatively low rate of molecular positivity can be attributed to the inclusion of patients who were already receiving antiretroviral therapy (ART). Since the primary goal of ART is to suppress viral replication to undetectable levels, a significant portion of the known HIV-positive population in our cohort likely had suppressed viral loads. Therefore, the RNA results reflect both newly diagnosed treatment-naïve cases and those under clinical follow-up with effective treatment outcomes. This outcome aligns with the UNAIDS 95-95-95 targets, specifically the third “95,” which aims for viral suppression in treated patients.

Numerous studies have demonstrated that a low CD4/CD8 ratio may result either from persistently low CD4 counts or from excessive CD8 counts, both of which lead to immune activation and are associated with an increased risk of serious non-AIDS events [15]. Mounting evidence suggests that the CD4/CD8 ratio may serve as a valuable indicator for monitoring HIV infection; however, some unanswered questions and areas of debate remain. During ART, a low CD4/CD8 ratio is associated with immunosenescence and inflammatory markers. It has also been linked to several potential mechanisms, including bacterial translocation, HIV persistence, and chronic latent coinfections [15]. Increased immunosenescence in the general population is reflected in CD8 cell expansion. In individuals with an inverted CD4/CD8 ratio (<1), CD8 cells have been shown to display shorter telomeres and to express senescence markers such as CD28 loss and oligoclonal expansion of cytomegalovirus-specific CD8+ T cells [16]. In our patient cohort, although absolute CD4 T-cell counts did not differ significantly between men and women, the CD4/CD8 ratio was higher in women than in men. When comparing absolute CD4 T-cell counts and CD4/CD8 ratios before treatment and at 3 months after treatment, both parameters showed an increase following therapy. Elevated CD4 T-cell counts and higher CD4/CD8 ratios during ART indicate reduced chronic inflammation and improved immunological restoration [17]. Rapid initiation of ART not only improves immunological recovery in individuals but also has significant public health implications. A recent modeling study from Türkiye demonstrated that early initiation of ART could markedly reduce both HIV incidence and AIDS-related mortality, while also increasing the likelihood of achieving the UNAIDS 95-95-95 targets [12]. Therefore, the increases observed in our patients may be considered indicators of a positive response to treatment.

“In Türkiye, recent epidemiological data indicate a shift toward younger age at HIV diagnosis: annual incidence has increased most rapidly among 15–19-year-olds (e.g., 13% among males and 11% among females) [18], while newly diagnosed cases are increasingly in younger age groups overall [19,20]. These trends underscore the need to tailor HIV testing and prevention strategies to youth populations.” Previous reports from Türkiye and neighboring countries have consistently shown that HIV infection is more common among males, a pattern often linked to behavioral, social, and epidemiological determinants [21]. The higher male prevalence observed in our study is therefore in agreement with long-standing national surveillance data and published academic reports, which describe a persistent male-to-female ratio of approximately 5:1. Although the overall HIV prevalence in Türkiye remains relatively low (0.1–0.3%), the number of newly diagnosed cases has increased markedly in recent years, a trend largely driven by infections within the male population [20].

This study has several limitations. First, the study population was heterogeneous, including individuals undergoing routine pre-operative screening, patients with suspected HIV infection, and patients under ART follow-up. This heterogeneity may have influenced the observed HIV prevalence, with a potential underestimation due to the inclusion of screening populations and a possible overestimation due to clinically suspected cases. Second, HIV RNA analysis included both newly diagnosed patients and individuals currently receiving ART, which may limit the interpretability of the reported RNA positivity rate. Therefore, the reported rates should be interpreted with caution, as they reflect a mixed clinical population rather than a single, well-defined cohort.

## 5. Conclusions

Having accurate information on the prevalence and risk factors of HIV/AIDS is essential for countries to develop effective health policies in the fight against this disease. Identifying which populations have a higher HIV prevalence enables health authorities to recognize at-risk groups, intervene for early diagnosis and treatment, and design more targeted and efficient public health strategies. Moreover, understanding the dynamics of viral spread and associated risk factors allows the timely implementation of preventive measures and facilitates the early detection of increases in AIDS cases.

In summary, our findings are consistent with our previous single-center study, yet the larger four-year cohort revealed a higher overall prevalence (0.25%) compared with 2022 (0.14%) [8]. Both studies confirmed the immune benefits of ART through improvements in CD4 T-cell counts and CD4/CD8 ratios. Taken together, these results indicate a gradual increase in HIV prevalence in our region, particularly among younger age groups and males. Although still relatively low, this upward trend represents a potential public health concern, emphasizing the need to strengthen prevention and early diagnosis strategies.

## Figures and Tables

**Table 1:** Distribution of demographic data and Anti-HIV results according to gender and years.

	Number of Individuals Tested (%)	Anti-HIV		*p* ^K^
		Negative, *n* (%)	Positive, *n* (%)	
Overall	271,367 (100) *	270,673 (99.75) *	694 (0.25) *	
**Gender**				
Men	118,896 (43.8) **	118,298 (99.5) *	598 (0.5) *	**<0.001**
Women	152,471 (56.2) **	152,375 (99.9) *	96 (0.1) *	
**Years**				
2020	43,021 (15.9) **	42,915 (99.2) *	106 (0.2) *	0.175
2021	54,838 (20.2) **	54,685 (99.7) *	153 (0.3) *	
2022	77,586 (28.6) **	77,372 (99.7) *	214 (0.3) *	
2023	95,922 (35.3) **	95,701 (99.8) *	221 (0.2) *	

*; Row percentage, **; Column percentage, K; Chi Square; Statistically significant results are written in bold.

**Table 2:** Distribution of individuals tested who underwent HIV-RNA testing according to gender, years, and age.

	Number of (Individuals Tested) (%)	HIV-RNA		*p* ^K^
		Negative (%)	Positive (%)	
Overall	2207 (100) *	1508 (68.30) *	699 (31.70) *	
**Gender**				
Men	1707 (77.3) **	1101 (64.50) *	606 (35.50) *	**<0.001**
Women	500 (22.7) **	407 (81.40) *	93 (18.60) *	
**Years**				
2020	435 (19.7) **	117 (26.9) *	318 (73.1) *	**<0.001**
2021	385 (17.4) **	161 (41.8) *	224 (58.2) *	
2022	592 (26.8) **	210 (35.5) *	382 (64.5) *	
2023	795 (36.1) **	211 (26.5) *	584 (73.5) *	
**Age Distribution**				
<18	22 (1.0) **	1 (4.5) *	21 (95.5) *	
18–24	208 (9.4) **	70 (33.7) *	138 (66.3) *	
25–34	553 (25.1) **	207 (37.4) *	346 (62.6) *	
35–44	579 (26.2) **	196 (33.9) *	383 (66.1) *	**<0.001**
45–54	462 (20.9) **	144 (31.2) *	318 (68.8) *	
55–64	248 (11.2) **	58 (23.4) *	190 (76.6) *	
>65	135 (6.1) **	23 (17.0) *	112 (83.0) *	

*; Row percentage, **; Column percentage, K: Chi Square; Statistically significant results are written in bold.

**Table 3:** Multivariate logistic regression analysis of factors associated with HIV-RNA positivity.

	OR	%95 CI	*p*
Gender (Men vs. Women)	2.41	1.89–3.08	**<0.001**
Age (An increase of 1 each year)	0.97	0.96–0.98	**<0.001**
Years (2020–2023)	1.05	0.98–1.12	0.147

OR: Odd ratio; Statistically significant results are written in bold.

**Table 4:** CD4 T cell count and CD4/CD8 ratio of individuals with positive HIV-RNA tests according to gender.

Gender (*n*, %)	T Cells	
	CD4+ Count (Cells/μL)	CD4+/CD8+
Men (*n* = 606, 86.7%)	444.0 (284.0–731.0)	0.52 (0.34–0.98)
Women (*n* = 93, 13.3%)	561.5 (320.5–706.5)	0.71 (0.38–1.02)
***p*** *	0.960	**<0.001**

*: Mann–Whitney U test; Statistically significant results are written in bold.

**Table 5:** T cell characteristics of HIV-RNA-positive patients.

T-Cells		Mean ± SD of T-Cells		*p* *
		Before ART	After ART	
CD4+ T cell count (cells/μL)		465.0 (290.25–733.25)	537.0 (365.25–828.25)	**<0.001**
CD4+/CD8+ ratio		0.51 (0.30–0.86)	0.70 (0.44–1.16)	**<0.001**
Men	CD4+ T cell count (cells/μL)	444.0 (282.0–732.5)	502.0 (405.5–885.5)	**<0.001**
	CD4+/CD8+ ratio	0.50 (0.29–0.84)	0.68 (0.43–1.14)	**<0.001**
Women	CD4+ T cell count (cells/μL)	561.5 (318.8–725.8)	612.0 (452.8–801.8)	0.060
	CD4+/CD8+ ratio	0.71 (0.38–1.02)	0.82 (0.52–1.30)	0.184

ART: antiretroviral therapy; *: Wilcoxon test; Statistically significant results are written in bold.

## Data Availability

All datasets are generated within this manuscript.
